# Artificial intelligence in traditional medicine: policy and governance strategies

**DOI:** 10.2471/BLT.24.292888

**Published:** 2025-09-16

**Authors:** Sameer Pujari, Rajeshwari Singh, Goh Cheng Soon, Tanuja Nesari, Ricardo Ghelman, Yu Zhao, Kanika Kalra, Shada Alsalamah, Richelle George, Shyama Kuruvilla, Alain Labrique

**Affiliations:** a Department of Data, Digital Health, Analytics and AI, Health Systems Division, World Health Organization, Avenue Appia 20, 1211 Geneva 27, Switzerland.; b Traditional and Complementary Medicine Division, Ministry of Health, Kuala Lumpur, Malaysia.; c Institute of Research and Teaching in Ayurveda, Jamnagar, India.; d Brazilian Academic Consortium for Integrative Health, São Paulo, Brazil.; e Information Systems Department, College of Computer and Information Sciences, King Saud University, Riyadh, Saudi Arabia.; f Global Traditional Medicine Centre, World Health Organization, Jamnagar, India.

Traditional, complementary and integrative medicine, as defined in the new World Health Organization (WHO) global traditional medicine strategy,^1^ encompasses a spectrum of health-care practices with distinct yet interrelated approaches. The definition includes traditional medicine rooted in cultural beliefs, complementary medicine used alongside conventional medicine and integrative medicine, which combines conventional and complementary therapies focusing on holistic and person-centred care.^2^

In this article, we examine the current landscape, identify key obstacles and propose strategies for addressing policy and governance challenges for leveraging the potential of artificial intelligence (AI) to advance traditional medicine systems.

AI is emerging as a key enabler in traditional medicine, supporting advancements in patient-centred care, personalized interventions, diagnostics, drug research and the digitization of traditional knowledge.^3^^,^^4^ AI technologies are advancing stakeholder capacities across four domains: people (end users), practitioners, practices and interventions. For end users, AI-powered applications and wearable devices provide personalized health insights and real-time monitoring,^5^ while practitioners benefit from diagnostic support and treatment planning tools.^6^ At the practice level, regarding operational flows and data management systems, AI facilitates the digitization of traditional medicine-based resources and the creation of structured, accessible knowledge databases.^7^ In terms of interventions, AI enables the design of personalized treatment plans by combining the principles of traditional medicine with clinical data in biomedicine, as seen in phytochemical profiling^8^ and Ayurgenomics, that is, the integration of Ayurvedic principles with genomics for translational medicine.^9^ These examples show that AI enhances clinical trial methods through predictive modelling and multimodal health data analysis.

Despite notable advances, integrating AI into traditional medicine poses critical challenges, including the need for high-quality, context-specific data; robust ethical, legal and regulatory frameworks; as well as effective interdisciplinary collaborations.^3^^,^^4^^,^^8^^,^^10^ Addressing these challenges is essential to responsibly harness the potential of AI in enhancing traditional medicine safety, mitigating bias, managing regulatory complexities and ensuring data security to prevent harm in traditional medicine.

## Transformative potential 

AI is at the forefront of the digital transformation of traditional medicine, offering three key opportunities.

The first opportunity regards clinical and health-care applications. AI is increasingly advancing into traditional medicine to enhance diagnostic precision, personalized treatment and holistic care. Machine learning supports syndrome classification in traditional Chinese medicine, such as Yang-Xu and Yin-Xu,^6^ and Ayurgenomics in Ayurveda^9^ through questionnaire-based analysis. In acupuncture, AI combined with ultrasound imaging improves needle placement by accurately distinguishing between muscle and nerve structures in real-time.^4^ Deep learning approaches enhance tongue and facial diagnostics by converting subjective assessments into quantifiable metrics.^6^ AI-powered clinical decision support systems generate personalized herbal prescriptions using symptom profiles, genomics and traditional diagnostic indicators like pulse patterns. Additionally, AI-enabled virtual assistants provide personalized health education, supporting disease prevention and self-care.^3^ Deep learning also facilitates image and/or text-based diagnostics and the development of knowledge graphs, while large language models, although still in an early stage of development in traditional medicine, show promise in diagnostic support, prescription generation and knowledge extraction.^3^^,^^4^

The second opportunity is the development of research, innovation and knowledge systems. Examples of AI-driven research and drug discovery include standardization of relevant data in traditional Chinese medicine,^8^ and the digitization of Ayurvedic, Siddha and Unani formulations by India’s Traditional Knowledge of Digital Library.^11^

The third opportunity is that of governance and health system strengthening. AI contributes to governance and health system strengthening by supporting policy design, strategic planning and regulation in traditional medicine. AI-driven electronic platforms like India’s Ayush Grid,^7^ and similar initiatives in Japan and Republic of Korea, strengthen health systems by improving access to traditional medicine through telemedicine and remote consultations.^12^

## Regulatory and governance challenges

Integrating AI into traditional medicine poses several regulatory and governance challenges.

First, integration of AI introduces regulatory complexities and legal accountability.^3^ Integrating AI into traditional medicine’s diverse therapies and practices introduces complexities in navigating health-care regulations.^8^^,^^10^ Determining responsibility in cases involving AI-related errors or adverse outcomes can be complex, requiring clear legal frameworks. Traditional medicine practitioners must be well versed in these legal aspects to ensure that their health-care practices are both ethical and compliant with the law.^10^

Second, digitizing traditional knowledge raises ethical concerns regarding intellectual property, biodiversity conservation, and equitable access and benefit-sharing.^11^ Ethical issues such as algorithmic bias, cultural erosion and lack of contextual sensitivity must be addressed to ensure responsible and respectful AI adoption.^4^^,^^10^

The third challenge is regarding data privacy and security.^3^^,^^4^ Traditional medicine encompasses sensitive, holistic health data, including medical, lifestyle, emotional health and sometimes genetic profiles. Patient data collection and analysis must adhere to global data protection laws, such as the General Data Protection Regulation (European Union), *Lei Geral de Proteção de Dados* (Brazil), Health Insurance Portability and Accountability Act (United States of America) and Personal Information Protection Law (China), to ensure the ethical and transparent handling and protection of personal health information.^13^

Fourth, the scarcity of standardized data is another challenge. Traditional medicine faces inconsistent terminologies and limited electronic medical records, which impede data integration and analysis. Developing foundational infrastructure, including standardized terminologies and electronic medical records, is essential. However, these steps are complicated by the diversity of traditional medicine practices, which span multiple regions and traditions, making it difficult to establish a unified global framework for data collection and use.^3^^,^^8^

Fifth, as AI is incorporated into traditional medicine, safeguarding a compassionate, human-centred patient-provider relationship is essential.^8^ Traditional medicine practitioners should use AI to support empathetic care by freeing up time for more meaningful patient interactions, rather than replacing them.^4^ Clear communication about the role of AI and obtaining informed consent are crucial to maintaining trust and transparency.^3^^,^^4^ Research is needed to understand patient perceptions, build trust and ensure alignment with the holistic values of traditional medicine. The integration of AI should be guided by ethical practices that reinforce, rather than undermine, the therapeutic and relational strengths central to traditional medicine.^4^^,^^10^

The last challenge is global and multistakeholder collaboration. Effective governance requires collaboration across governments, industries, communities and practitioners. International efforts, such as WHO’s Global Centre for Traditional Medicine and the Global Initiative on AI for Health Topic Group on AI and traditional medicine, are essential for harmonized and inclusive policy development on this issue.

## Policy strategies 

The responsible adoption of AI in traditional medicine calls for well-defined policy strategies that respect cultural heritage, promote equity and uphold ethical standards. Key considerations include regulatory oversight, intellectual property protection and data privacy. A three-pronged approach is essential to foster a transparent, responsible and inclusive environment for AI integration in traditional medicine.

First, regulatory frameworks must be adapted to address the challenges posed by AI technologies in traditional medicine. These should be comprehensive yet flexible, ensuring safety, accountability and quality assurance while accommodating the diversity of traditional practices.

Second, AI-driven digitization of these traditional practices raises critical concerns related to intellectual property and biodiversity. The use of AI to catalogue and analyse traditional knowledge often linked to local ecosystems and species heightens the risk of biopiracy and misappropriation. Frameworks such as the Nagoya Protocol and Traditional Knowledge of Digital Library^11^ are essential for safeguarding Indigenous rights and preventing biopiracy. Concurrently, robust data governance policies must prioritize informed consent, encryption and algorithmic transparency, aligning with global AI and data ethics standards.

Third, building the capacity of traditional medicine practitioners through targeted training and infrastructure support are crucial for effective AI implementation. Ensuring contextual relevance and cultural sensitivity in AI applications is key to maintaining trust and ensuring equitable benefits. To foster innovation and enhance the adoption of AI-based digital solutions in traditional medicine, policy frameworks must prioritize research grounded in scientific rigour and support targeted capacity-building for traditional medicine practitioners.

Strengthening collaboration across the health, technology, environment and education sectors is also essential. International organizations such as WHO, the World Intellectual Property Organization, the International Telecommunication Union, and the International Organization for Standardization play a pivotal role in facilitating knowledge exchange and establishing global standards.

These efforts are critical to ensuring that AI technologies are ethically, effectively and equitably integrated into traditional medicine systems to enhance accessibility, promote environmental stewardship and improve health outcomes.

To support the responsible use of AI in traditional medicine, we suggest policy-makers take the following actions. First, establish clear legal frameworks, by defining regulatory responsibilities and accountability mechanisms for AI use in traditional medicine to ensure legal clarity for developers, practitioners and institutions. Second, ensure ethical, cultural and ecological integrity, by safeguarding traditional practices through fair intellectual property practices and developing AI systems that are culturally sensitive, ecologically informed and free from bias. Third, strengthen data governance, by implementing robust data protection policies aligned with global standards and emphasizing informed consent, privacy and transparency in AI applications. Fourth, standardize data and infrastructure, by supporting the development of harmonized terminologies, interoperable data systems and electronic health records to facilitate cross-border collaboration and system integration. Fifth, build the capacity of traditional medicine practitioners through scientifically rigorous research and training to ensure practitioners are empowered to responsibly and appropriately leverage AI for the benefit of their patients. Finally, promote inclusive collaboration by encouraging multistakeholder and international partnerships to ensure that AI integration in traditional medicine is ethical, participatory and responsive to local and global health priorities.

Integrating AI into traditional medicine presents a unique opportunity to enhance its relevance, accessibility and effectiveness, thereby supporting broader recognition of its value within global health systems. Addressing key policy, regulatory and ethical challenges through inclusive governance, strong legal frameworks and respect for cultural and ecological values is essential. Responsibly applied, AI can bridge traditional wisdom with modern technology to advance equitable and sustainable health improvements in the twenty-first century.
